# Development and Characterization of a High-CBD Cannabis Extract Nanoemulsion for Oral Mucosal Delivery

**DOI:** 10.3390/ijms262311525

**Published:** 2025-11-27

**Authors:** Kifah Blal, Georgette Maroukian, Anna Shapira, Shiri Procaccia, David Meiri, Ofra Benny

**Affiliations:** 1 Department of Oral and Maxillofacial Surgery, Faculty of Dental Medicine, Hebrew University of Jerusalem, Jerusalem 9112102, Israel; dr.k.blal@mail.huji.ac.il; 2 School of Pharmacy, Faculty of Medicine, Hebrew University of Jerusalem, Jerusalem 9112001, Israel; georgette.maroukian@mail.huji.ac.il; 3 Laboratory of Cancer Biology and Natural Drug Discovery, Faculty of Biology, Technion—Israel Institute of Technology, Haifa 3200003, Israel; annasha@technion.ac.il (A.S.); shirip@technion.ac.il (S.P.)

**Keywords:** cannabidiol, cannabis extract, nanoemulsion, oral mucosal delivery, oral lichen planus, graft-versus-host disease, head and neck squamous cell carcinoma, immunomodulation

## Abstract

The cannabidiol (CBD)-rich cannabis extract CAN296 shows anti-inflammatory and anticancer activity relevant to oral lichen planus (OLP), oral graft-versus-host disease (oGVHD), and oral squamous cell carcinoma (OSCC), but its high lipophilicity limits aqueous dispersion. This study developed a stable Tween-based nanoemulsion optimized for oral mucosal delivery. Ethanol-dissolved CAN296 was nanoemulsified using a 1% Tween/Span system. Physical stability was visually assessed; droplet size and morphology were examined by dynamic light scattering (DLS) and transmission electron microscopy (TEM); and wettability was measured by static contact angle (SCA). Additional evaluations included temperature stability (25 °C vs. 4 °C), in vitro release using a dialysis membrane, and scanning electron microscopy (SEM) of membrane-associated droplets. Nanoemulsions with ≥80% Tween 80 incorporated CAN296 up to 800 µg/mL, clear at 400 µg/mL, and uniformly turbid at 800 µg/mL. DLS and TEM confirmed spherical nanoscale droplets, and SCA indicated favorable cohesion and wettability. Stability was maintained for 30 days at 4 °C. Dialysis studies demonstrated strong membrane association with limited diffusion, supported by SEM visualization of membrane-bound droplets. The Tween-dominant (≥80%) nanoemulsion stably incorporated CAN296 up to 800 µg/mL, demonstrated nanoscale uniformity, improved 4 °C stability, and strong membrane retention under static conditions, suggesting potential for localized oral delivery.

## 1. Introduction

Cannabis-derived extracts rich in cannabidiol (CBD) have significant therapeutic potential in immune-mediated and oncologic oral diseases due to their anti-inflammatory, immunomodulatory, and pro-apoptotic effects [1,2,3,4,5,6]. These extracts suppress CD4^+^ and CD8^+^ T-cell activation, reduce pro-inflammatory cytokines such as TNF-α and IFN-γ, and inhibit cytotoxic mediators including granzyme B, perforin, and Fas ligand [7,8,9]. CBD-rich extracts also induce selective apoptosis in malignant oral epithelial cells in HNSCC models [10,11,12,13,14,15], and the addition of cannabichromene (CBC) enhances this activity [4].

The biological activity of cannabis extracts is primarily mediated through the endocannabinoid system (ECS), which regulates immune function, inflammation, and epithelial homeostasis [16,17,18,19,20]. The ECS includes CB1/CB2 receptors, endogenous ligands (AEA, 2-AG), associated enzymes, and extended targets such as orphan GPCRs, TRP channels, and PPARs [21,22,23,24,25,26,27]. Our previous work demonstrated that CAN296 and CBD:CBC formulations exert potent immunomodulatory and pro-apoptotic effects in OLP, oGVHD, and HNSCC models [4,9].

OLP and oGVHD are chronic T-cell–mediated inflammatory disorders with impaired quality of life and recognized malignant potential [28,29]. OLP presents with reticular, erosive, or plaque-like lesions [30] and has a global prevalence of 0.5–4% [31]. Both OLP and oGVHD are associated with a risk of transformation to OSCC, which is influenced by lesion type and duration [32,33]. GVHD occurs following allogeneic HSCT and results from donor T-cell–mediated tissue damage [34,35], with oral lesions resembling OLP [34,35] and similar malignant potential [36,37]. Their shared pathophysiology involves activation of CD4^+^/CD8^+^ T cells, secretion of IFN-γ and TNF-α, and cytotoxic injury through FasL, perforin, and granzyme B [28,38,39,40,41]. Chronic inflammation and epithelial instability contribute to carcinogenic risk [42,43,44], linking both disorders to HNSCC, a major global malignancy [45,46,47].

Current treatments rely on topical corticosteroids such as clobetasol and triamcinolone [48,49], but long-term use can cause mucosal atrophy, candidiasis, and reduced efficacy [50,51,52,53]. Calcineurin inhibitors (tacrolimus, cyclosporine) inhibit IL-2–mediated T-cell activation [54,55] but may cause systemic toxicity [56,57], and chronic disease often requires prolonged therapy [58,59]. Existing treatments do not address the risk of malignant transformation.

Cannabinoids, particularly those derived from CBD-type extracts, have demonstrated antitumor activity in several cancer models, including oral squamous cell carcinoma [5,6]. Alongside CBD, other phytocannabinoids such as THC, as well as terpenes, contribute to therapeutic efficacy through synergistic “entourage” interactions [60,61,62]. Cannabinoids influence multiple tumor-related pathways, including angiogenesis and tumor-microenvironmental signaling [63,64,65,66,67,68,69]. Importantly, CBD-type extracts selectively target malignant epithelial cells while sparing healthy tissues [4,5], making them attractive candidates for chronic inflammatory oral disorders with malignant potential.

Tween 80 and Span 80 were selected as the surfactant blend because both are pharmaceutically approved, non-ionic excipients commonly used in oral and buccal drug delivery and exhibit low irritation potential at the concentrations applied. Their complementary hydrophilic–lipophilic balance (HLB) values allow fine-tuning of interfacial stabilization for highly lipophilic cannabis extracts, while maintaining compatibility with ethanol, which was required to solubilize the extract. Preliminary screening with alternative mucosal excipients—including polysorbates, poloxamers, lipid-based carriers, and mucin-interacting polymers—did not yield stable emulsions under ethanol-containing conditions or failed to accommodate the required extract load. For these reasons, Tween 80/Span 80 provided the most suitable and pharmaceutically acceptable platform for nanoemulsion optimization in this study.

We hypothesized that incorporating the CBD-rich cannabis extract CAN296 into a Tween-dominant nanoemulsion system (≥80% Tween 80) would result in superior physicochemical performance compared with the crude extract oil, specifically by improving nanoscale droplet uniformity, wettability, and mucosal membrane interaction. These properties are expected to enhance local bioavailability and stability, thereby overcoming the limitations of the native lipophilic extract. Accordingly, this study aimed to develop and characterize a stable, ethanol-compatible CBD-rich nanoemulsion optimized for oral mucosal application. The optimized formulation will serve as the basis for subsequent biological assays to evaluate its immunomodulatory and cytotoxic potential in oral inflammatory and precancerous conditions, including OLP, oGVHD, and early-stage HNSCC.

## 2. Results

### 2.1. Cannabis Nanoemulsions with up to 800 µg/mL Load Are Stabilized by 1% Surfactant Containing ≥80% Tween

**Figure 1:**
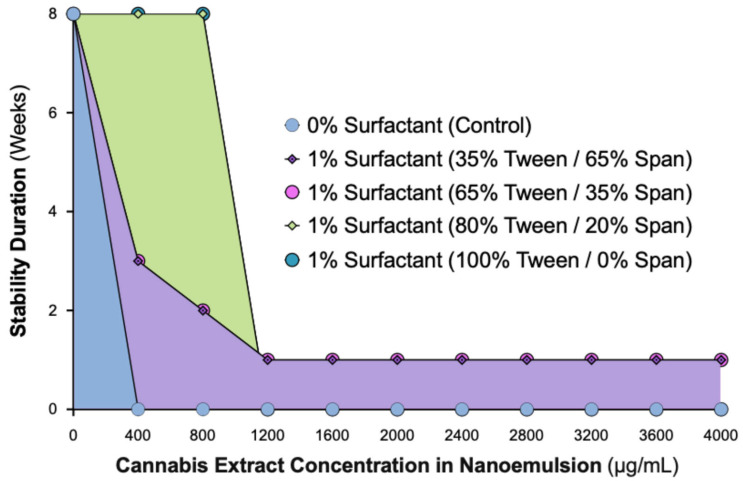
Stability of 1% Tween/Span CAN296 nanoemulsions across varying Tween ratios and extract concentrations (0–4000 µg/mL). Physical stability was visually assessed over 8 weeks. Symbols represent surfactant compositions: 0% surfactant control (blue circles), 35% Tween/65% Span (purple diamonds), 65% Tween/35% Span (pink circles), 80% Tween/20% Span (green diamonds), and 100% Tween/0% Span (teal circles). Shaded regions indicate the stability range for each formulation group: blue shading represents the 0% surfactant control, purple shading corresponds to 35–65% Tween/Span mixtures, and green shading corresponds to the 80% Tween formulation.

Initial attempts using the crude, non-formulated cannabis extract (without surfactant) resulted in immediate phase separation, confirming that the native extract cannot form a stable aqueous dispersion. Therefore, subsequent experiments focused on determining whether a surfactant-based system could achieve and maintain nanoscale stability. To define the optimal formulation for long-term physical stability, we evaluated the influence of varying Tween:Span ratios using a constant 1% surfactant system, with cannabis extract concentrations up to 4000 µg/mL. Each formulation was visually monitored over an 8-week observation period (Figure 1).

Formulations without surfactant showed immediate phase separation at all concentrations. Nanoemulsions containing 35% or 65% Tween exhibited only short-term stability, with a turbid (milky) appearance and eventual separation. In contrast, formulations containing ≥80% Tween (80:20 or 100:0 Tween:Span) demonstrated sustained physical stability throughout the observation period. At 400 µg/mL, these nanoemulsions remained optically clear, while 800 µg/mL formulations were uniformly turbid without phase separation. Concentrations ≥1200 µg/mL showed visible oil separation across all surfactant ratios, indicating that the system’s solubilization capacity had been exceeded.

These results confirm that a 1% surfactant system requires at least 80% Tween 80 to achieve long-term stability for cannabis concentrations up to 800 µg/mL. Beyond this threshold, the system becomes unstable, establishing the formulation limits under the tested conditions. This guided our selection of the 80% Tween formulation for subsequent analyses.

### 2.2. Dynamic Light Scattering Analysis Confirms Stability Limit of 800 µg/mL Cannabis in 1% Surfactant Nanoemulsions with ≥80% Tween

**Figure 2:**
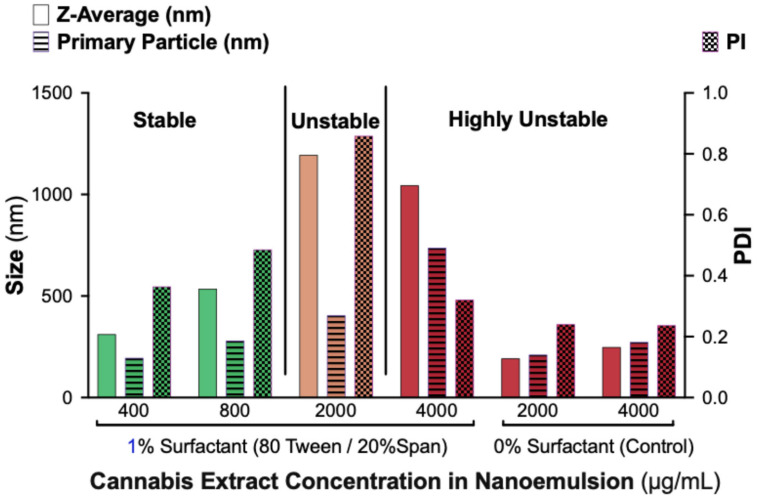
Effect of CAN296 load on particle size and PDI in nanoemulsions containing 1% surfactant (80% Tween/20% Span). Bar plots show Z-average size (unfilled bars), primary particle size (horizontal-line bars), and PDI (pattern-filled bars) for formulations with 400–4000 µg/mL CAN296. The left Y-axis indicates particle size (nm), and the right Y-axis indicates PDI. Samples are grouped into surfactant-containing formulations (**left**) and surfactant-free controls (**right**). Classification zones (“Stable,” “Unstable,” “Highly Unstable”) reflect visual appearance and DLS metrics.

To characterize nanoemulsion stability in terms of droplet size distribution, we performed DLS analysis on formulations containing 1% surfactant (80% Tween/20% Span) at extract concentrations of 400, 800, 2000, and 4000 µg/mL. Surfactant-free controls (0% Tween/Span) were also evaluated at 2000 and 4000 µg/mL (Figure 2).

Formulations containing 400 and 800 µg/mL cannabis displayed nanoscale droplet populations with moderate polydispersity, consistent with physical stability. In contrast, formulations containing 2000 and 4000 µg/mL showed markedly increased droplet sizes and high PDIs, indicating loss of nanoscale structure and early aggregation. Control samples without surfactant exhibited small but highly heterogeneous, multi-population size distributions, reflecting rapid phase separation and poor colloidal uniformity.

Together, these results confirm that surfactants are essential for generating homogeneous nanoemulsions and that the maximum cannabis load stably accommodated by a 1% surfactant system containing ≥80% Tween 80 is approximately 800 µg/mL. The DLS findings support the visual stability observations and define the formulation limits of the system.

### 2.3. Transmission Electron Microscopy (TEM) Reveals Progressive Particle Homogeneity with Increasing Tween Content

**Figure 3:**
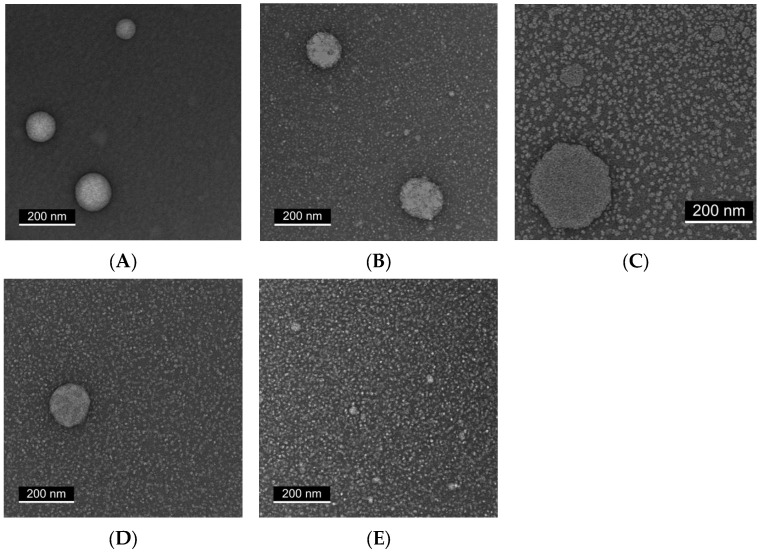
TEM images showing that ≥80% Tween promotes formation of uniform, monodisperse droplets in CAN296 nanoemulsions (400 µg/mL) at different Tween:Span ratios. Representative micrographs are shown for: (**A**) 0% surfactant (control), (**B**) 35% Tween/65% Span, (**C**) 65% Tween/35% Span, (**D**) 80% Tween/20% Span, and (**E**) 100% Tween. All samples were imaged under identical magnification; white scale bar = 200 nm.

To visualize the morphological characteristics of the nanoemulsions, representative samples containing 400 µg/mL cannabis extract and varying Tween:Span ratios were examined by TEM (Figure 3). All formulations contained 1% total surfactant except for the surfactant-free control.

In the absence of surfactant (Panel A), large and irregular oil droplets with pronounced heterogeneity were observed. Formulations containing 35% or 65% Tween (Panels B and C) showed smaller droplets but remained heterogeneous, with irregular aggregates and variable particle sizes.

By contrast, formulations containing ≥80% Tween (Panels D and E) displayed progressively improved morphological uniformity. The 80% Tween sample produced more spherical, monodisperse droplets, while the 100% Tween formulation showed a dense and uniform nanoscale droplet distribution without visible aggregation. These observations are consistent with the DLS findings, confirming that higher Tween content promotes homogeneous nanoemulsion formation at a fixed extract concentration.

### 2.4. Static Contact Angle (SCA) Measurements Reveal Nonlinear Wettability with Maximal Cohesion at 800 µg/mL

**Figure 4:**
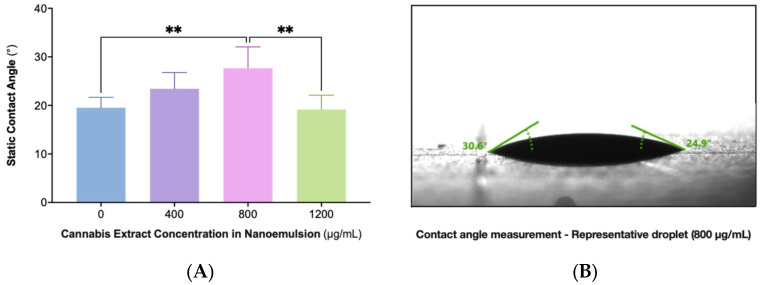
SCA measurements of CAN296 nanoemulsions at increasing extract concentrations. (**A**) Contact angle values for formulations containing 0, 400, 800, and 1200 µg/mL, measured on glass substrates using the sessile-drop method (5 µL droplets; mean ± SD, n = 6; *p* < 0.01, one-way ANOVA with Tukey’s test). Asterisks (**) mark comparisons that reached statistical significance (*p* < 0.01). (**B**) Representative droplet image of the 800 µg/mL formulation. Abbreviations: SCA, static contact angle; DSA, drop shape analyzer; CAN296, CBD-rich cannabis extract.

Surface wettability was evaluated across nanoemulsions containing 0, 400, 800, and 1200 µg/mL cannabis extract using static contact angle measurements on glass substrates (Figure 4). The formulations demonstrated a concentration-dependent change in spreading behavior, with the 800 µg/mL sample exhibiting the highest contact angle and therefore the greatest droplet cohesion. Lower (0 and 400 µg/mL) and higher (1200 µg/mL) extract concentrations showed lower angles indicative of increased spreading. Statistical analysis confirmed that the 800 µg/mL formulation differed significantly from the other concentrations. These findings demonstrate a nonlinear relationship between extract load and interfacial behavior, with maximal cohesion observed at 800 µg/mL. A representative droplet image for this formulation is shown in Panel B.

### 2.5. Enhanced Stability of Nanoemulsions at 4 °C Compared to Room Temperature After 30 Days

**Figure 5:**
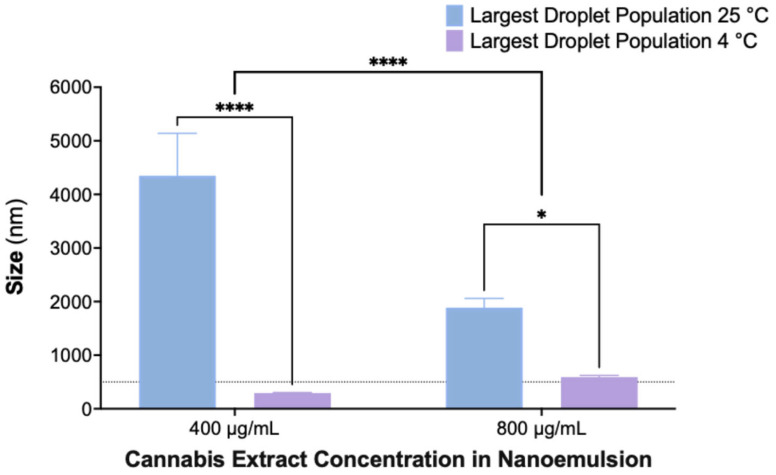
Largest detected droplet size (nm) of CAN296 nanoemulsions after 30 days of storage at room temperature (25 °C, blue) and 4 °C (purple). DLS measured the largest droplet population in formulations containing 400 and 800 µg/mL CAN296 (mean ± SD, n = 3). Statistical analysis was performed using two-way ANOVA with Šidák’s multiple comparisons, including the interaction between temperature and extract concentration. All asterisks represent the simple main effect of temperature (25 °C vs. 4 °C) within each cannabis concentration. No post-hoc pairwise comparisons were performed for the concentration factor. Significance levels: * *p* = 0.0259; **** *p* < 0.0001. The dashed line marks 500 nm as a nanoscale reference. Abbreviations: CAN296, CBD-rich cannabis extract; DLS, dynamic light scattering.

To assess the effect of storage temperature on nanoemulsion stability, formulations containing 400 and 800 µg/mL cannabis extract were stored for 30 days at either room temperature (25 °C) or under refrigeration (4 °C), and droplet size distributions were analyzed by DLS (Figure 5). The largest detected droplet population was used as an indicator of aggregation and coalescence.

Nanoemulsions stored at 4 °C maintained droplet sizes within the nanoscale range, whereas those stored at 25 °C exhibited pronounced droplet growth, indicating temperature-dependent destabilization. Statistical analysis showed significant effects of both temperature and extract concentration, with refrigerated samples displaying substantially smaller droplet populations at both concentrations. These findings demonstrate that storage at 4 °C effectively reduces coalescence and preserves nanoemulsion integrity over time.

### 2.6. Significant In Vitro Retention of Cannabis Extract Nanoemulsion on Dialysis Membrane Suggests Mucoadhesive Potential

**Figure 6:**
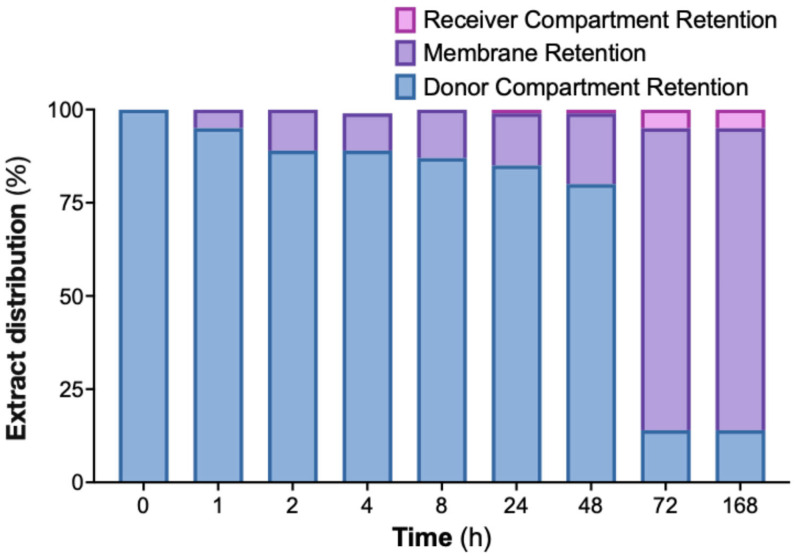
Distribution of CAN296 during in vitro release from a nanoemulsion (800 µg/mL) using a dialysis device (Pur-A-Lyzer™ Mini 6000, 1 kDa). Samples were collected at predefined time points (0–168 h). Bars show the percentage of extract in the donor compartment (blue), receiver compartment (pink), and membrane-retained fraction (purple; calculated as donor loss minus receiver gain). Statistical analysis was performed using one-way ANOVA (F(2,27) = 21.27, *p* < 0.0001, R^2^ = 0.6118). Abbreviation: CAN296, CBD-rich cannabis extract.

To evaluate the interaction between the nanoemulsion and a semipermeable membrane, an in vitro dialysis experiment was performed over 168 h using a formulation containing 800 µg/mL of cannabis extract (Figure 6). The extract-loaded nanoemulsion was placed in the donor compartment, while a blank nanoemulsion was added to the receiver compartment. Samples from both compartments were collected at predefined time points and analyzed for CBD content.

Throughout the experiment, the donor compartment showed a progressive decrease in extract content, whereas the receiver compartment showed only a modest, gradual increase. The discrepancy between donor depletion and receiver accumulation indicated that a substantial fraction of the extract was associated with the dialysis membrane. This membrane-retained portion is shown in Figure 6 and accounts for the majority of the extract not recovered in the receiver phase.

Together, these findings demonstrate that the nanoemulsion exhibits strong membrane interaction under the test conditions, consistent with prolonged surface residence and suggesting prolonged membrane association under static in vitro conditions.

### 2.7. Scanning Electron Microscopy (SEM) Visualization of Nanoemulsion Aggregates on Dialysis Membrane Surface

**Figure 7:**
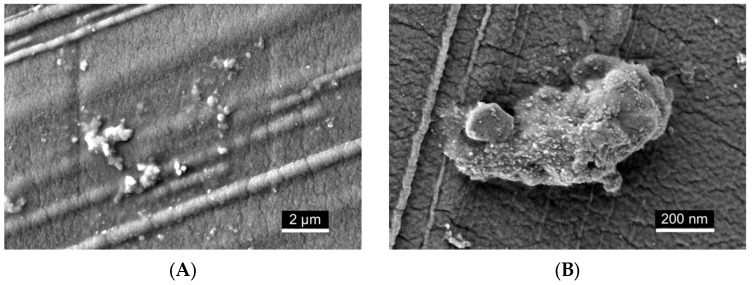
SEM imaging of CAN296 nanoemulsion material on the dialysis membrane surface. (**A**) Lower-magnification micrograph (white scale bar = 2 µm) showing spherical droplets and small clusters distributed across the membrane. (**B**) Higher-magnification micrograph (white scale bar = 200 nm) revealing densely packed submicron aggregates with irregular morphology. These observations are descriptive and do not indicate mucoadhesion. Abbreviations: CAN296, CBD-rich cannabis extract; SEM, scanning electron microscopy.

To visualize the localization of cannabis extract nanoemulsion on the dialysis membrane surface, an additional dialysis capsule was prepared under the same conditions described in Section 4.8, using formulations containing either 400 or 800 µg/mL of cannabis extract (Figure 7). After 24 h of incubation, the capsule was disassembled, and the membrane was gently removed. It was then rinsed with distilled water, air-dried, and sputter-coated with a thin layer of iridium (2–3 nm) for SEM imaging.

SEM imaging qualitatively revealed deposition of nanoemulsion material on the membrane surface. At lower magnification (Figure 7A), scattered spherical droplets and small clusters were visible across the surface. At higher magnification (Figure 7B), visually denser regions of submicron aggregates were observed, forming irregular, closely packed structures. The intrinsic pore structure of the membrane was not resolved under the imaging conditions, likely due to dehydration and sputter coating, which may have obscured finer surface features. These observations are descriptive and based on qualitative visual assessment.

## 3. Discussion

Formulating a CBD-rich cannabis extract into a physically stable nanoemulsion required careful optimization of surfactant composition, extract concentration, and energy input to achieve nanoscale uniformity and mucosal compatibility. The optimized formulation, containing 1% total surfactant (≥80% Tween 80) and ethanol as a cosolvent, demonstrated long-term physical stability, consistent droplet distribution, favorable wettability, and characteristics suitable for oral mucosal delivery.

In contrast to most cannabinoid nanoemulsions, which typically require high-energy homogenization and elevated surfactant levels to achieve sub-200 nm clarity, this study demonstrates that a robust, ethanol-compatible system can be prepared with only 1% total surfactant using a Tween-dominant composition. Systematic optimization showed that ≥80% Tween 80 was required to stabilize extract concentrations up to 800 µg/mL. Concentrations below 1% failed to maintain nanoscale stability in ethanol-containing systems, whereas higher surfactant levels offered no additional benefit and were avoided to reduce mucosal irritation. This highlights a low-surfactant, biocompatible platform for localized oral delivery.

Cannabis-derived formulations are most commonly administered orally, yet edible and ingestible products show low and variable bioavailability (6–20%) due to first-pass metabolism and cannabinoid lipophilicity [70,71,72]. Ethanol-based sublingual tinctures partially bypass hepatic metabolism but may cause mucosal irritation and show limited local retention [70,73,74]. In contrast, the nanoemulsion platform developed here enhances solubility, wettability, and mucosal adhesion, supporting its use for localized treatment of chronic inflammatory and precancerous oral conditions.

The nanoemulsion was prepared using a MICCRA high-shear disperser, providing sufficient mechanical energy to form nanoscale droplets while preserving extract integrity. This moderate-energy method avoids the elevated surfactant requirements, thermal load, and potential phytochemical degradation associated with high-pressure or ultrasonic homogenization [75,76,77,78]. However, the study did not include a direct comparison with high-energy techniques. Future work will therefore evaluate high-energy–processed formulations to determine whether further reductions in droplet size or improvements in kinetic stability can be achieved.

Initial attempts with the crude, non-formulated cannabis extract revealed that it could not be maintained as a stable aqueous dispersion, exhibiting immediate phase separation even under high shear. Consequently, direct comparative testing between the extract alone and the formulated nanoemulsion was not feasible. The study, therefore, focused on evaluating the physicochemical feasibility and performance of surfactant-based nanoemulsions as a prerequisite for subsequent biological testing.

A Tween-dominant surfactant system enabled long-term nanoscale stability with extract concentrations up to 800 µg/mL. Given that CAN296 contains ~55% CBD, this corresponds to ~440 µg/mL of CBD, exceeding reported effective in vitro concentrations for inflammatory and malignant oral epithelial models [4,9,10,11,12,13,14,15]. Nanoemulsions remained optically clear at 400 µg/mL and uniformly turbid but stable at 800 µg/mL, whereas ≥1200 µg/mL exceeded the solubilization capacity of the system. DLS and TEM confirmed nanoscale droplets with moderate polydispersity at ≤800 µg/mL and heterogeneous aggregation at higher loads.

SCA measurements showed that both 400 and 800 µg/mL formulations exhibited contact angles within the optimal range for oral mucosal wetting [79]. The 800 µg/mL sample demonstrated the highest cohesion, supporting concentration-dependent effects on spreading behavior relevant to mucosal retention.

Storage temperature strongly influenced stability: nanoemulsions stored at 4 °C maintained nanoscale droplets for 30 days, whereas samples at 25 °C exhibited pronounced coalescence (>4000 nm). These findings are consistent with previous cannabinoid nanoemulsions, which also demonstrate improved physicochemical stability under refrigeration, driven by reduced kinetic instability and suppressed Ostwald ripening [80,81,82].

A limitation of this stability assessment is the absence of freeze–thaw, light-exposure, and extended refrigerated storage studies. Such conditions are essential for defining shelf-life and robustness, and future work will incorporate these stress tests.

In vitro release testing showed that approximately 14% of the loaded extract diffused into the receiver compartment over 168 h. Because the dialysis system lacks the hydrodynamic, enzymatic, and turnover characteristics of the oral cavity, the ~14% release should not be interpreted as therapeutic availability but rather as diffusion through a restrictive 1 kDa membrane under static conditions. Published in-vitro studies using CBD-rich extracts report biological activity in oral epithelial and inflammatory models at CBD-equivalent concentrations of ~5–50 µg/mL and 20–100 µg/mL in malignant models [4,9,10,11,12,13,14,15], indicating that physiologically relevant exposure may occur even with partial release. Nevertheless, confirming therapeutic relevance requires dedicated mucosal transport studies and cell-based assays using the nanoemulsion itself. A further methodological limitation relates to the receiver phase: a blank nanoemulsion was used to maintain surfactant equilibrium, but this medium does not mimic human saliva, which contains ions, proteins, mucins, and enzymes that influence droplet stability and cannabinoid diffusion. Future studies will therefore employ standardized simulated saliva media to more accurately assess release behavior and mucosal availability under physiologically relevant conditions.

The study did not evaluate phytochemical stability after homogenization or storage. Cannabinoids can undergo oxidation, isomerization, or decarboxylation in ethanol-containing or shear-intensive environments. Future UHPLC or LC–MS/MS analysis will determine whether the nanoemulsion protects cannabinoids from degradation over time.

Mucosal biocompatibility must be established before clinical translation. Although Tween 80 is widely used in buccal formulations, concentrations approaching 1% may cause transient irritation in susceptible tissues. The formulation also contains 25% ethanol, comparable to commercial oral rinses such as Listerine^®^ (~26.9% *v*/*v*), which produce only transient exposure without long-term adverse effects [83]. However, repeated exposure to ethanol ≥20% may exacerbate dryness or epithelial disruption in conditions such as erosive OLP or oGVHD. Future studies will therefore assess epithelial viability, barrier integrity, and irritation potential in ethanol-reduced and ethanol-free formulations.

SEM imaging confirmed the presence of nanoemulsion droplets and aggregated structures on the dialysis membrane surface, supporting qualitative surface interaction. However, these findings should not be interpreted as evidence of true mucoadhesion, as mucoadhesive oral delivery systems require prolonged residence and controlled release demonstrated in dedicated models [84,85,86]. The study did not include control formulations—such as surfactant-free ethanolic dispersions or alternative surfactant systems—necessary to determine whether the observed retention is formulation-specific or simply nonspecific adsorption to the membrane. Moreover, the SEM analysis was qualitative and lacked quantitative metrics such as surface coverage, aggregate size distribution, or relative membrane occupancy. Future work will incorporate comparative controls and quantitative image analysis to distinguish formulation-dependent deposition from nonspecific adsorption and to evaluate interaction with physiologically relevant mucosal substrates.

## 4. Materials and Methods

### 4.1. Phytocannabinoid Extraction and Sample Preparation

Air-dried Type III high-CBD cannabis CAN296 was obtained from a licensed Israeli medical cannabis distributor. Inflorescences were extracted in ethanol and decarboxylated by heating at 130 °C. The resulting extract was dissolved in dimethyl sulfoxide (DMSO; Sigma-Aldrich, St. Louis, MO, USA) and stored at −20 °C.

Phytocannabinoid profiling was performed using a UHPLC system coupled with a Q Exactive Focus Hybrid Quadrupole-Orbitrap mass spectrometer (Thermo Scientific, Bremen, Germany), as previously described [4]. Identification and absolute quantification of cannabinoids were achieved using analytical standards and external calibration curves (Thermo Scientific, Bremen, Germany), following previously established protocols [87].

### 4.2. Formulation Preparation

Cannabis nanoemulsions were prepared by dissolving the CAN296 extract in 25% ethanol, which served as a cosolvent to aid dispersion of the lipophilic extract. The extract solution was then emulsified with 1% surface-active agent composed of Tween 80 and Span 80 (Sigma-Aldrich, St. Louis, MO, USA) at varying ratios. Cannabis concentrations ranged from 0 to 4000 µg/mL in 400 µg/mL increments. After 5 min of stirring, the mixtures were homogenized using a MICCRA D-9 disperser (MICCRA GmbH, Heitersheim, Germany) at 11,000 rpm for 3 min.

### 4.3. Stability Evaluation of Nanoemulsions by Visual Inspection

Nanoemulsion stability was evaluated through systematic visual inspection under consistent ambient lighting conditions. Samples were stored in clear, sealed vials at room temperature (~25 °C) and assessed weekly over 8 weeks (days 7, 14, 21, 28, 35, and 42). Each formulation was observed for signs of turbidity, creaming, sedimentation, and phase separation. Nanoemulsions were classified as Stable if they remained uniform (transparent or turbid) with no visible separation; Unstable if they exhibited droplet aggregation or partial separation with increasing turbidity or layering; and Highly Unstable if they underwent complete separation or sedimentation within the first week. These assessments served as a primary tool to evaluate formulation robustness across different surfactant ratios and cannabis concentrations.

### 4.4. Dynamic Light Scattering Analysis

Droplet size, PDI, and colloidal stability of cannabis nanoemulsions were assessed using DLS. Formulations contained 1% surfactant (80% Tween 80/20% Span 80) with 800, 2000, and 4000 µg/mL cannabis, along with surfactant-free controls. Measurements were performed at 25 °C on a Zetasizer ZS XPLORER (Malvern Panalytical Ltd., Malvern, UK) in triplicate. Z-average, PDI, and intensity-weighted distributions were recorded and analyzed with ZS XPLORER software, version 3.3.0.42. Results were interpreted in conjunction with visual stability data to classify nanoemulsions as stable, unstable, or highly unstable. It should be noted that DLS provides an intensity-weighted average that can overrepresent larger droplet populations in polydisperse systems; therefore, the measured Z-average and PDI values reflect relative rather than absolute particle sizes.

### 4.5. TEM Imaging

Morphological analysis of nanoemulsion particles was performed using TEM. Samples were adsorbed onto Formvar-carbon-coated copper grids (EMS, 200 mesh) and negatively stained with 2% uranyl acetate to enhance image contrast. After staining, the grids were allowed to air-dry before imaging. TEM was performed using a JEOL JEM-1400 Plus transmission electron microscope (JEOL Ltd., Tokyo, Japan) operated at 100 kV. High-resolution images were acquired using a Gatan Orius 600 CCD camera, allowing for detailed visualization of droplet morphology, particle size, and aggregation patterns across various surfactant formulations.

### 4.6. SCA Measurement

Wettability of the CBD-rich cannabis nanoemulsion was evaluated using a KRÜSS Drop Shape Analyzer (DSA; KRÜSS GmbH, Hamburg, Germany) equipped with ADVANCE software (v1.11.2.25901). Glass microscope slides (Thermo Scientific, Germany) were cleaned with ethanol, rinsed with deionized water, and air-dried before use. A 5 µL droplet of each formulation (0, 400, 800, and 1200 µg/mL) was dispensed with a microsyringe, and SCA values were measured immediately using the sessile drop method. Left- and right-angle measurements were automatically averaged, with six replicates per sample under ambient laboratory conditions (~25 °C).

### 4.7. Phytocannabinoid Identification and Quantification

Phytocannabinoid analyses were performed using a Thermo Scientific UHPLC system coupled with a Q ExactiveTM Focus Hybrid Quadrupole-Orbitrap mass spectrometer (Thermo Scientific, Bremen, Germany). Identification and absolute quantification of phytocannabinoids were performed using analytical phytocannabinoid standards and Cannabis samples at preset concentrations, as previously described [87].

### 4.8. In Vitro Release Kinetics Using a Dialysis Membrane System

The release of CBD from the nanoemulsion (800 µg/mL) was evaluated using a dialysis kit (Pur-A-Lyzer Mini 6000, 1 kDa molecular weight cutoff; Sigma-Aldrich, Merck KGaA, Darmstadt, Germany) with a 1 kDa pore membrane. The cannabis-loaded nanoemulsion was placed inside the membrane chamber, and a blank nanoemulsion was added to the outer compartment. Twelve sealed vials were incubated at room temperature under constant agitation. At ten predefined time points (0, 0.5, 1, 2, 4, 8, 24, 48, 72 h, and 7 days), samples were collected separately from the internal (donor) and external (receiver) compartments. UHPLC was used to analyze all samples for CBD concentration (Section 4.7), and release values were corrected based on the extract’s CBD content (~50%).

### 4.9. SEM Imaging

Samples were examined using an Extra-High-Resolution Scanning Electron Microscope (Magellan 400 L, FEI Company, Hillsboro, OR, USA) after plasma sputter-coating with a thin iridium layer (2–3 nm) to enhance surface conductivity. Each membrane sample was imaged in three representative regions at varying magnifications to assess surface morphology and potential pore obstruction. SEM images were processed using ImageJ software (version 1.54g, NIH, USA) for qualitative visualization. Uniform brightness and contrast adjustments were applied to all images to enhance the visibility of surface features. Thresholding was used to improve the contrast between droplets and the membrane background, without compromising image integrity.

### 4.10. Statistical Analysis

All statistical analyses were conducted using GraphPad Prism version 10.6.1 for macOS (GraphPad Software, San Diego, CA, USA). Inferential statistical analyses were performed where applicable to assess differences between formulations and conditions. SCA data, storage stability measurements (droplet size), and in vitro release profiles were analyzed using one-way or two-way analysis of variance (ANOVA) with appropriate post hoc tests (Tukey’s or Šidák’s multiple comparisons), as specified. Data are presented as mean ± SD of at least three independent measurements unless otherwise noted. Visual inspection, DLS measurements, and TEM imaging were interpreted descriptively to evaluate qualitative trends in nanoemulsion clarity, temporal stability, droplet size distribution, and polydispersity.

## 5. Conclusions

This research established a Tween-dominant nanoemulsion capable of stabilizing a robust concentration of CBD-rich cannabis extract. This optimized system remains stable under refrigeration, exhibits favorable wettability and membrane retention, and provides a physically stable, ethanol-compatible platform for oral mucosal delivery of cannabis extract. Future research should evaluate mucosal biocompatibility and in vivo retention to advance translational development for OLP, oGVHD, and early-stage HNSCC.

## Data Availability

The data presented in this study are available upon request from the corresponding author due to ethical restrictions on donor confidentiality.

## Abbreviations

The following abbreviations are used in this manuscript:

2-AG	2-arachidonoylglycerol
ANOVA	Analysis of variance
AEA	N-arachidonoylethanolamine (anandamide)
CAN296	CBD-rich cannabis extract (Type III strain)
CB1	Cannabinoid receptor type 1
CB2	Cannabinoid receptor type 2
CBC	Cannabichromene
CBD	Cannabidiol
CD4^+^	Cluster of differentiation 4 helper T cells
CD8^+^	Cluster of differentiation 8 cytotoxic T cells
DLS	Dynamic light scattering
DMSO	Dimethyl sulfoxide
DSA	Drop shape analyzer
ECS	Endocannabinoid system
GVHD	Graft-versus-host disease
HNSCC	Head and neck squamous cell carcinoma
HSCT	Hematopoietic stem cell transplantation
IFN-γ	Interferon gamma
IL-2	Interleukin-2
oGVHD	Oral graft-versus-host disease
OLP	Oral lichen planus
OSCC	Oral squamous cell carcinoma
PDI	Polydispersity index
PPAR	Peroxisome proliferator-activated receptor
PPARγ	Peroxisome proliferator-activated receptor gamma
SCA	Static contact angle
SD	Standard deviation
SEM	Scanning electron microscopy
TAC	Tacrolimus
TEM	Transmission electron microscopy
THC	Δ^9^-tetrahydrocannabinol
TNF-α	Tumor necrosis factor alpha
TRP	Transient receptor potential
UHPLC	Ultra-high-performance liquid chromatography
